# Effects of Protein Hydrolysate from Silkworm (*Bombyx mori*) pupae on the C2C12 Myogenic Differentiation

**DOI:** 10.3390/foods12152840

**Published:** 2023-07-26

**Authors:** Hyeong-Seok Kang, Ji Hye Park, Joong-Hyuck Auh

**Affiliations:** Department of Food Science and Technology, Chung-Ang University, Anseong 17546, Republic of Korea

**Keywords:** edible insects, C2C12 cell differentiation, muscle differentiation, silkworm pupae

## Abstract

This study investigated the effects and active compounds of silkworm pupae, an edible insect, on C2C12 muscle differentiation. The protein of silkworm pupae was extracted using sonication after defatting with hexane. Subsequently, the extract was rehydrated using Alcalase to obtain a protein hydrolysate. The silkworm pupae protein hydrolysate effectively promoted C2C12 myogenic differentiation without cytotoxicity. Subsequently, the hydrolysate was fractionated into four subfractions using preparative high-performance liquid chromatography (Prep-HPLC). Subfraction 1 was the most effective in promoting C2C12 myogenic differentiation and significantly upregulated the expression of myoblast transcription factors, 1.5-fold of myoblast determination protein 1 (MyoD), 2-fold of myogenin, and 3-fold of myosin heavy chain (MyHC). Liquid chromatography–tandem mass spectrometry (LC-MS/MS) and multivariate statistical analysis were used to identify the active peptides in silkworm pupae responsible for the observed effects; then, dipeptides and essential amino acids, such as isoleucine (Ile), valine (Val), and methionine (Met), were identified. In addition, Val, Ile, and two dipeptides underwent quantification to determine the potential bioactive peptides that enhanced C2C12 myogenic differentiation. This study suggests that the peptides from silkworm pupae could be used as a nutraceutical to enhance muscle growth.

## 1. Introduction

In the food industry, proteins and peptides are used as ingredients for nutritional intake and processed food, but they are also a source of functional food [1]. However, the future of our food supply is precarious. Beyond climate change and global population growth, which is expected to reach 9 billion by 2050, overexploitation and depletion of natural resources are critical factors that may jeopardize food security in the coming years [2]. Regarding food availability, the growing demand for meat and the limited amount of agricultural land available for livestock prompted the search for alternative proteins [3]. Food scientists turned to plant-based proteins, such as soy protein, as animal-derived proteins are failing to satisfy the requirements of the global population and dietary demand [4]. Most plant-based proteins have a low essential amino acid content, are often deficient in one or more specific amino acids, and are more difficult to digest than animal proteins [4].

Considering this, the benefits of edible insects as an alternative source of animal proteins are gaining increased attention. Edible insects are more sustainable than animals in terms of land use and greenhouse gas emissions [5]. Additionally, they have high protein contents, 40% on average, which is as much as animal proteins [6]. Moreover, positive research results were generated for incorporating insect protein into low-nutrient foods, such as snacks, pasta, and bread [4]. Snacks with the addition of 10~20% of mealworm larvae (*Tenebrio molitor*) showed an increased protein content, better digestibility, and similar textural properties to their insect-free counterparts [7].

In South Korea, seven kinds of insects were approved for use in food [8], and their bioactive peptides are gaining momentum as a source of functional food [9]. For example, the peptides from B. mori have α-glucosidase inhibitory activity [10], and cricket (*Gryllodes sigillatus*)-, mealworm (*T. molitor*)-, and locust (*Schistocerca gregaria*)-derived peptides have anti-inflammatory and antioxidant effects [11]. Silkworm (*B. mori*) pupae, a by-product of the silk industry, are mainly utilized as animal feed, while they are also widely used as a food ingredient in Asian countries such as China, India, Thailand, and Korea [12]. Silkworm proteins were previously associated with in vitro antioxidant properties, while enzymatic hydrolysates of silkworm proteins were noticed to generate higher antioxidant hydrolysates [13,14]. However, no studies were conducted related to muscle growth with edible insects sources.

Murine-origin C2C12 myoblast cells are widely used to examine muscle growth [15] due to easy expansion and culture conditions [16]. Initially, the cells exist as myoblasts that fuse in a serum-deprived environment to form multinucleated myotubes [17]. Various pathways, such as the extracellular signal-regulated kinase (ERK) 1/2, p38, and phosphoinositide-3-kinase (PI3K), are involved in muscle growth. The ERK 1/2 signaling pathway is required for myoblast proliferation [18], and the p38 and PI3K signaling pathways promote myoblast differentiation. P38 affects the expression of MyoD, an essential factor in myoblast differentiation, and subsequently initiates the expression of another differentiation transcription factor, myogenin [19]. Moreover, activated PI3K converts the plasma membrane lipid phosphatidylinositol-4,5-bisphosphate to phosphatidylinositol-3,4,5-trisphosphate and activates Akt, which, in turn, activates the mammalian target of rapamycin (mTOR), MyoD, and myogenin sequentially [20]. MyoD is involved in the early step of differentiation when myoblasts are sufficiently proliferated. Subsequently, myogenin is involved in further differentiation. When myoblasts are fused into a myotube, MyHC, a major structural protein of myotube, can be observed [21]. MyoD and myogenin are most highly expressed after 3 days of differentiation, and MyHC has the highest expression after 5 days of differentiation [19].

Therefore, the present study explored the effect of protein hydrolysates from silkworm pupae on C2C12 myogenic differentiation using an immunofluorescence assay. Prep-HPLC fractionated SP, and the increase in the expression of the transcription factors of muscle differentiation-MyoD, myogenin, and MyHC by each subfraction was observed. Active metabolites were screened and identified via LC-MS-based metabolomic analysis using multivariate statistical analysis.

## 2. Materials and Methods

### 2.1. Materials and Reagents

Silkworm (*B. mori*) pupae were obtained from Insect Vision (Yangju, Republic of Korea) and stored in a deep freezer (−50 °C) to preserve the specimens until use. Liquid chromatography (LC)-grade water was purchased from Honeywell (Charlotte, NC, USA). Guaranteed reagent (GR)-grade methanol (MeOH) and extra pure (EP)-grade hexane were purchased from Daejung Chemicals & Metals (Daejung, Siheung, Republic of Korea). Mass spectrometry (MS)-grade water, acetonitrile, and formic acid were purchased from Thermo Fisher Scientific (Waltham, MA, USA). Dulbecco’s phosphate-buffered saline (DPBS) and Dulbecco’s modified Eagle medium (DMEM) were purchased from Biowest (Cholet, France), and PBS was purchased from Bio-Rad Laboratories (Hercules, CA, USA). Horse serum, penicillin–streptomycin, and trypsin were purchased from Gibco (Grand Island, NY, USA). L-Isoleucine and L-Valine were purchased from Sigma-Aldrich (St. Louis, MO, USA).

### 2.2. Defatting and Protein Extraction

As in the previous study [22], four steps were followed to prepare insect protein hydrolysate. Prior to the defatting process, the insect was comminuted using a traditional pestle and mortar. The comminuted insect was defatted with EP-grade hexane at 1:20 (*w*/*v*) to increase the protein yield. The mixture was stirred for 36 h, and the hexane was replaced every 12 h with filtering. Defatted insects were dried overnight at room temperature under a fume hood. Following the defatting, proteins were extracted via sonication (Sonics^®^ Vibra-Cell™ VCX 750 ultrasonic unit, Sonics & Materials, Inc., Newtown, CT, USA), on ice, at 20 kHz for 5 min, with 75% amplitude and pulsed every 3 s with an interval of 1 s. Defatted insects (12.5 g) and 200 mL of an aqueous solution of ascorbic acid (9.46 mM) were mixed in a ratio of 1:16. The extracts were filtered, freeze-dried, and then stored in a deep freezer (−50 °C) until use.

### 2.3. Protein Hydrolysis and Ethanol (EtOH) Precipitation

The crude extract was rehydrated with distilled water (DW), and 72 mU/g of Alcalase was added to obtain the insect protein hydrolysate. The reaction was carried out in a water bath at 55 °C for 4 h, and the resultant hydrolysate was centrifuged at 15,000× *g* for 30 min. The supernatants were freeze-dried and stored in a deep freezer (−50 °C) until use.

Insect protein hydrolysate was rehydrated with 50 mM of phosphate-buffered saline (PBS, pH 7.0) at a 50 mg/mL concentration to obtain the hydrophilic protein hydrolysate. EtOH was added, and the protein hydrolysate was precipitated at −20 °C for 3 h. The precipitated sample was centrifuged at 15,000× *g* for 40 min and the supernatants were concentrated using a rotary vacuum evaporator (Eyela, Tokyo, Japan) to remove EtOH. The concentrated samples were freeze-dried and stored in a deep freezer (−50 °C) until use.

### 2.4. SDS-PAGE

The protein degradation patterns of each pretreatment step were checked by sodium dodecyl sulfate–polyacrylamide gel electrophoresis (SDS-PAGE) using a 15% polyacrylamide running gel. The protein concentration was measured by the bicinchoninic acid (BCA) assay. The gel was stained and destained after electrophoresis (120 V, 100 min, Bio-Rad Laboratories). The gel image was captured using the Molecular Imager Gel Doc™ XR+ (Bio-Rad Laboratories).

### 2.5. Cell Culture

Murine-origin C2C12 (ATCC, Manassas, VA, USA) myoblast cells were seeded in a 10 cm cell culture dish at a cell density of 3 × 10^5^. C2C12 cells were maintained in the DMEM with 10% fetal bovine serum (FBS), 100 U/mL penicillin, and 100 µg/mL streptomycin. At a confluence of 95~100% [23], the medium was replaced with a differentiation medium (DMEM with 2% horse serum, 100 U/mL penicillin, and 100 µg/mL streptomycin). The cells were further incubated at 37 °C in a humidified atmosphere of 5% CO_2_.

### 2.6. Immunofluorescence Analysis

C2C12 cells were seeded in 12-well plates at 7 × 10^4^ cells/well and 24-well plates at 4 × 10^4^ cells/well and proliferated in growth media. The differentiation rate was monitored by immunocytochemistry. After 2 days of proliferation, the samples were dissolved in LC-grade water and treated simultaneously as the growth media was replaced with differentiation media. After 3 days, during which the myoblasts fused into myotubes, the cells were washed with PBS and fixed with 4% paraformaldehyde for 30 min at room temperature. After fixation, the wells were blocked with 3% bovine serum albumin (BSA) in PBS at 4 °C for 1 h. Subsequently, cells were treated with mouse anti-myosin-4 monoclonal antibody Alexa 488 (Invitrogen, Waltham, MA, USA) at a dilution of 1:100 in 3% BSA in PBS at 4 °C for 24 h to detect MyHC protein. Cells were washed with PBS and counterstained with 4′6-diamidino-2-phenylindole dihydrochloride (DAPI, 1:5000; Invitrogen) in PBS for 30 min at room temperature. Cells were again washed 3 times with PBS and stored at 4 °C in the presence of PBS [24]. Images were taken using an inverted fluorescence microscope, and the fusion index (number of nuclei incorporated into fused myotubes/total number of nuclei) was calculated to determine myotube fusion [25]. The differentiation-promoting effect and the fusion index were evaluated relative to the control group.

### 2.7. MTT Assay

The method described by [26] was slightly modified and optimized for this study. C2C12 cells were seeded in a 96-well plate at 1 × 10^4^ cells/well. After 12 h of incubation, the cells were treated for 20 h with SP dissolved in DW (50, 100, 200, 400, and 800 μg/mL). Subsequently, 20 µL of 1 mg/mL methyl thiazole tetrazolium (MTT) in DPBS was added and incubated for 4 h. After removing the medium, the formazan crystals were dissolved in 200 μL of dimethyl sulfoxide (DMSO, Samchun Chemical, Pyeongtaek, Republic of Korea). Absorbance was measured at 570 nm using a microplate reader (SpectraMax^®^ M2, Molecular Devices, San Jose, CA, USA). The cytotoxicity was evaluated relative to the cell viability of the control.

### 2.8. Western Blot Analysis

For this study, 3 × 10^5^ cells were seeded in a 6 cm cell culture dish and proliferated in a growth medium. At 95% confluence, the sample was treated at the same time that the growth medium was replaced with the differentiation medium, and the cells were differentiated for 3 days. Differentiation for 3 days was sufficient to observe MyoD, myogenin, and MyHC by immunochemical analysis. After 3 days, during which the myoblasts changed to myotubes, the cells were washed 3 times with PBS (Bio-Rad Laboratories), harvested with PBS supplemented with 0.1% protease inhibitor, and centrifuged at 15,000× *g* for 5 min at 4 °C. Afterward, the residue was lysed with lysis buffer (mammalian protein extraction buffer added with 1% protease inhibitor and 1% phosphatase inhibitor) for 15 min on ice and centrifuged at 15,000× *g* for 15 min at 4 °C. Supernatants were collected, and the BCA assay detected the protein concentration. Briefly, 40 μg of proteins were mixed with 4× Laemmli sample buffer (44.4% *v*/*v*, glycerol, 277.8 mM Tris–HCl pH 6.8, 10% 2-mercaptoethanol, 4.4% LDS, and 0.02% bromophenol blue) at a 4:1 ratio and boiled at 100 °C for 5 min. The mixtures were loaded onto 5% polyacrylamide stacking gel and 8% polyacrylamide running gel. Loaded proteins were transferred to a poly (vinylidene difluoride) membrane (PVDF membrane, Millipore, Billerica, MA, USA) and blocked with 5% BSA in TBST at room temperature for 1 h. The membrane was then treated with primary antibodies, anti-MyoD (1:1000; Invitrogen), anti-myogenin (1:1000; Santa Cruz Biotechnology, Dallas, TX, USA), and anti-MyHC (1:200; Cell Signaling, Danvers, MA, USA) for 2 days at 4 °C [27]. The membrane was washed with TBST and treated with a secondary antibody, anti-mouse (1:5000; Cell Signaling), for 3 h at room temperature. The proteins were visualized using EZ-Capture MG (ATTO, Tokyo, Japan) and quantified using the CS analyzer (ver. 3.0; ATTO).

### 2.9. Fractionation Using Preparative HPLC

SP was fractionated using a Prep-HPLC instrument (LC-Forte/R, YMC, Kyoto, Japan) equipped with an ODS C18 column (250 × 15 mm, 20 μm) [8]. The solvent system consisted of DW (solvent A) and MeOH (solvent B) at a 10 mL/min flow. The elution program was as follows: 0% B for 5 min and 30% B for 30 min for a total run of 30 min. All fractions were concentrated using a rotary vacuum evaporator (Eyela) to remove MeOH. Concentrates were freeze-dried and stored in a deep freezer (−50 °C) until use.

### 2.10. Identification of Active Metabolites by LC-MS/MS

To identify the active metabolites in SP-F1 that enhanced C2C12 myogenic differentiation, LC-MS/MS was performed using a Vanquish UHPLC apparatus coupled to an Orbitrap mass spectrometer (Q Exactive Plus MS, Thermo Fisher Scientific) equipped with a heated electrospray ionization (HESI) interface (Thermo Fisher Scientific). LC-MS/MS was performed using the mobile phases of solvent A (0.1% formic acid in water) and solvent B (0.1% formic acid in acetonitrile) at a 0.3 mL/min flow rate. The time program was as follows: 5% B for 3 min, 12% for 10 min, 30% for 14 min, 100% for 16 min, and 5% for 20 min, for a total run time of 20 min. The UV spectrum was measured from 200 to 400 nm. The analysis was performed in the positive-ion mode with the HESI source at a spray voltage of 3.80 kV. The scan range was 100–1500 *m*/*z*, and the capillary temperature was 320 °C. The sheath and auxiliary gas were 40 and 10 mL/min, respectively.

### 2.11. Quantitative Analysis

The amount of active amino acids was quantified using LC-MS/MS in the PRM mode. The LC-MS/MS was performed under the conditions mentioned above. A standard curve graph was plotted as the intensity area versus the concentration of each amino acid. The intensity area of each amino acid was measured using 5 mg/mL of SP-F1 and quantified using a standard curve. The concentration of the calibration points for each amino acid was analyzed in triplicate.

### 2.12. Data Processing and Statistical Analysis

LC-MS/MS data were processed using MZmine 2.0. Several steps were implemented, including raw data import, spectral filtering, peak detection, isotope classification, chromatographic alignment, gap filling, and data export [28]. Then, the SIMCA software (version 17.0; Umetrics, Umeå, Sweden) was used for multivariate statistical analysis. Triplicate results were expressed as the mean ± standard deviation. One-way analysis of variance (ANOVA) and Duncan’s test (*p* < 0.05) were performed using the Statistical Package for the Social Sciences software (SPSS, version 26; SPSS Inc., Chicago, IL, USA). To identify differences or similarities among experimental groups, principal component analysis (PCA) and orthogonal partial least squares discriminant analysis (OPLS-DA) were performed using the SIMCA software (Umetrics). The variable importance in the projection (VIP) value and *p*-value were used to compare the differences between the two groups.

## 3. Results

### 3.1. Yields of Each Pretreatment Process of Silkworm pupae (%)

Three pretreatment steps (extraction, enzyme-induced hydrolysis, and ethanol precipitation) were performed to isolate the protein hydrolysate from silkworm pupae. After protein extraction, hydrolysis, and EtOH precipitation, the yields were 84.2%, 74.7%, and 42.2%, respectively.

### 3.2. Degradation Patterns of Silkworm pupae Protein Hydrolysis by Different Pretreatments

**Figure 1:**
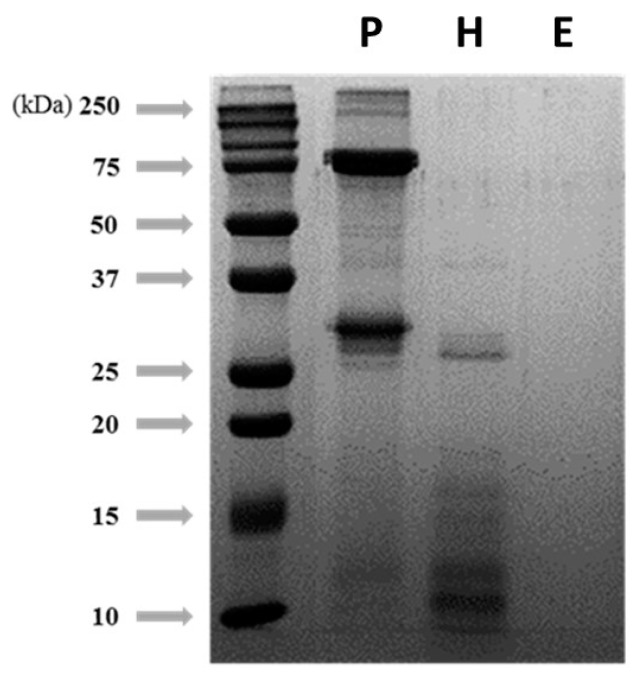
SDS-PAGE analysis for the degradation patterns of silkworm pupae protein hydrolysis by different treatment steps. Silkworm protein extract was hydrolyzed with Acalase (72 mU/g protein) for 4 h at 55 °C. P, crude protein extracts; H, protein hydrolysate by Acalase; and E, soluble proteins after ethanol precipitation.

SDS-PAGE was used to characterize the profile of silkworm pupae protein at each pretreatment step: extraction, enzyme-induced hydrolysis, and EtOH precipitation (Figure 1). The significant bands of the protein extract were located at 250, 75, and 30 kDa. After hydrolysis with Alcalase, the molecular weight distribution of the proteins mostly shifted below 25 kDa, and after EtOH precipitation, most of the proteins greater than 10 kDa significantly disappeared due to an enzymatic reaction.

### 3.3. Effect of Protein Hydrolysate from Silkworm pupae on C2C12 Myogenic Differentiation: Immunofluorescence Assay

The possible cytotoxicity of SP was assessed by treating C2C12 cells with a range of concentrations of SP and measuring cell viability using the MTT assay under the concentration range of 50~800 μg/mL. No cytotoxicity was observed.

**Figure 2:**
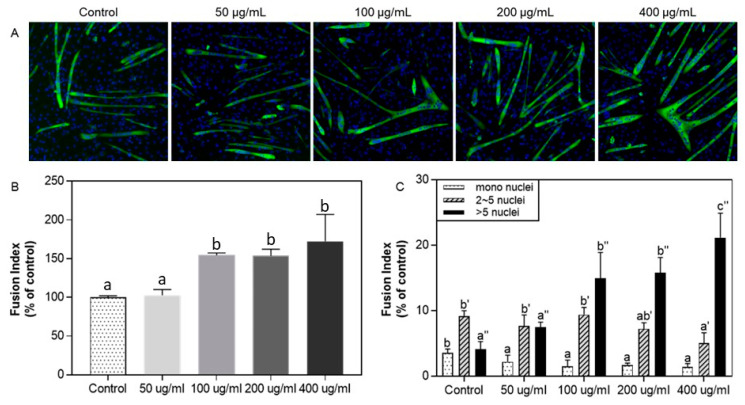
Effect of protein hydrolysate from silkworm pupae (SP) on C2C12 myogenic differentiation: immunofluorescence assay. (**A**) C2C12 cells immunostained with anti-MyHC and DAPI were treated with different concentrations of SP (50, 100, 200, and 400 μg/mL); (**B**) the effect of SP on the fusion index for myotube formation; and (**C**) the My-HC positive cells were classified to mononucleus, 2–5 nuclei, and >5 nuclei. Three independent data were expressed as mean ± SD. Different superscripts indicate a significant difference (*p* < 0.05).

The immunofluorescence assay confirmed SP’s effect on C2C12 cell differentiation. Treatment with silkworm pupae protein was extended to 50~400 μg/mL to verify its differentiation-promoting effect with the fusion index (Figure 2). The number of MyHC-positive myotubes increased above 100 μg/mL of SP even though no significant increase was observed between 200 and 400 μg/mL. Subsequently, the fusion index was assessed by calculating the number of MyHC-positive cells and classifying them as mononucleus, 2–5 nuclei, and more than 5 nuclei for each group [25]. As shown in Figure 2C, the mononucleus was the highest in the control, myotubes with 2–5 nuclei were highest in the control, and SP was at 50 μg/mL. Above a 100 μg/mL concentration, myotubes with > five nuclei were the highest.

### 3.4. Effect of Purified Protein Hydrolysate from Silkworm pupae on C2C12 Myogenic Differentiation by the Immunofluorescence Assay

**Figure 3:**
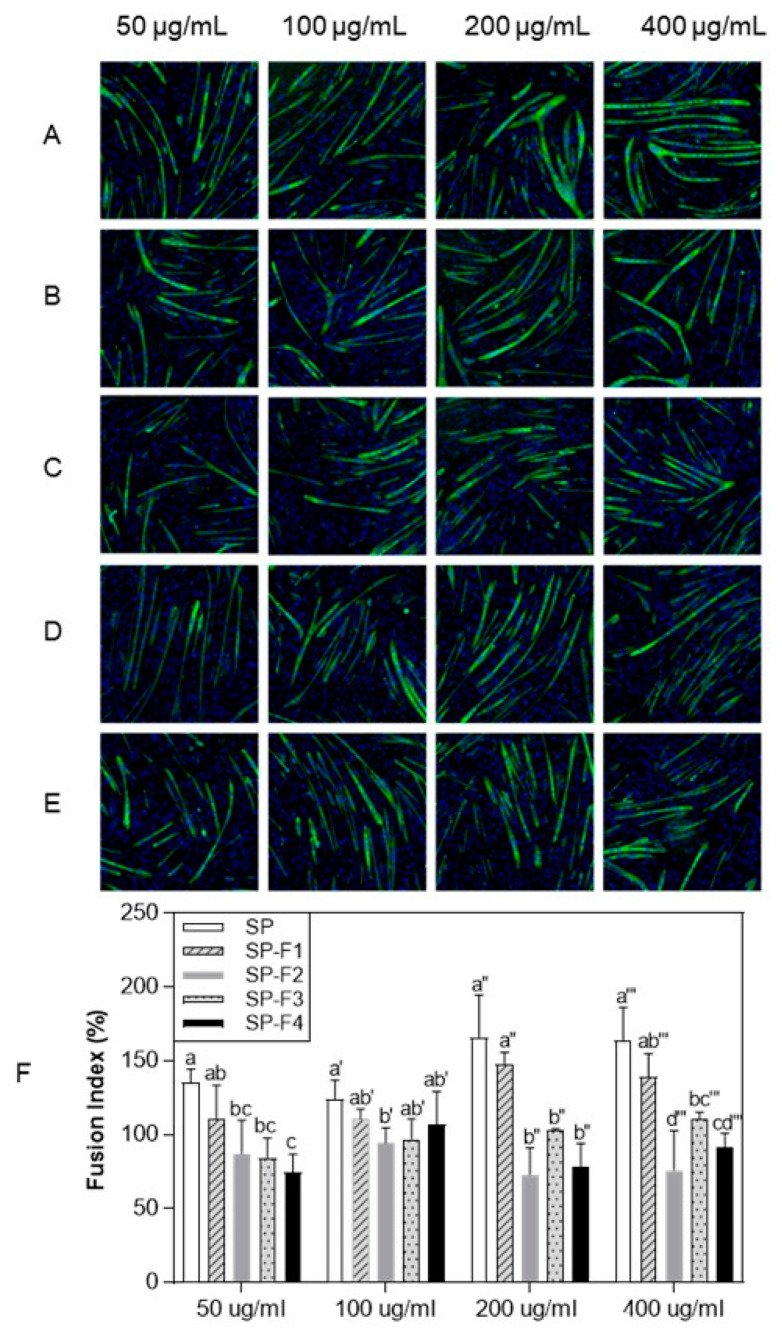
Effect of fractionated protein hydrolysate from silkworm pupae (SP-Fs) on C2C12 myogenic differentiation: immunofluorescence assay. C2C12 cells immunostained with anti-MyHC and DAPI were treated with different concentrations of SP-Fs (50, 100, 200, and 400 μg/mL). (**A**), control; (**B**), SP-F1; (**C**), SP-F2; (**D**), SP-F3; (**E**), SP-F4; and (**F**), the effect of SP-Fs on the fusion index for myotube formation. Three independent data were expressed as mean ± SD. Different superscripts indicate a significant difference (*p* < 0.05).

To identify the active peptides/compounds responsible, SP was fractionated into four subfractions by Prep-HPLC. The C2C12 myogenic differentiation-promoting activity of the four subfractions (SP-F1, SP-F2, SP-F3, and SP-F4) was observed by the same immunofluorescence method described above (Figure 3) and was indicated as the fusion index (Figure 3F). SP-F1 was most significantly active at 200 and 400 μg/mL concentrations compared to the other subfractions.

### 3.5. Effects of Silkworm pupae Protein Hydrolysate on the Expression of MyoD, Myogenin, and MyHC

**Figure 4:**
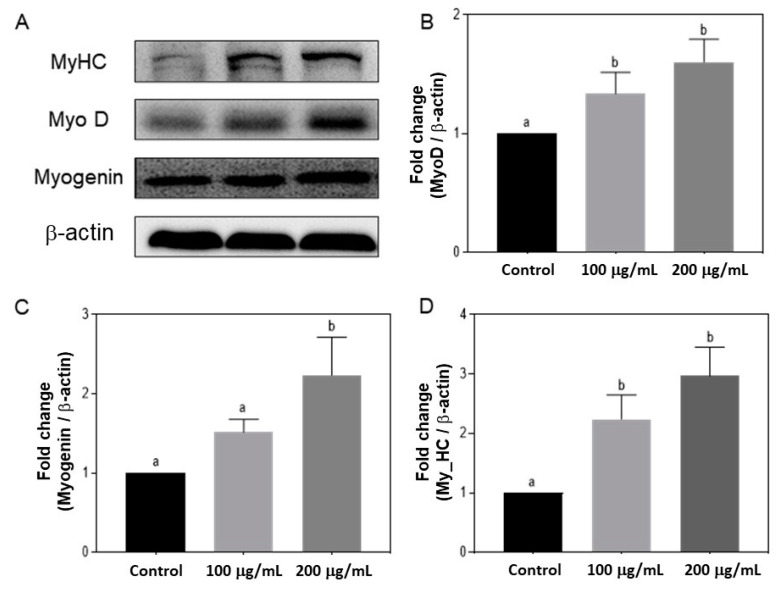
Effects of the subfraction 1 of silkworm pupae protein hydrolysate (SP-F1) on the expression of MyoD, myogenin, and MyHC of C2C12 myogenic differentiation. (**A**) Western blot analyses were performed to examine the expression of the myogenic regulatory factor proteins, namely MyoD (**B**), Myogenin (**C**), and MyHC (**D**), and their levels were quantified. Three independent data were expressed as mean ± SD. Different superscripts indicate a significant difference (*p* < 0.05).

Among the four subfractions, SP-F1 was most active in promoting C2C12 myogenic differentiation. Thus, the effect of SP-F1 on the transcription factors (MyoD, myogenin, and MyHC) expressed during muscle cell differentiation was observed using a Western blot assay (Figure 4). SP-F1 significantly increased the expression of all three transcription factors in a concentration-dependent manner (100 and 200 μg/mL). MyoD-positive cells exit the cell cycle during the myoblast differentiation process, whereas myogenin begins to be expressed to initiate differentiation, and MyHC is expressed as the cells differentiate and fuse into myotubes.

### 3.6. Metabolomic Analysis by Multivariate Statistical Analysis

To further identify the active peptides/compounds responsible for promoting C2C12 myogenic differentiation, LC-MS/MS analysis was performed. The distinctive compounds in each subfraction were separated through multivariate statistical analysis, and the four subfractions formed four separate groups in the PCA plot. SP-F4 was distinguished from SP-F1, SP-F2, and SP-F3 by principal component 1 (PC1, 48.4%), and SP-F1 and SP-F2 were distinguished from SP-F3 and SP-F4 by principal component 2 (PC2, 36.2%).

**Figure 5:**
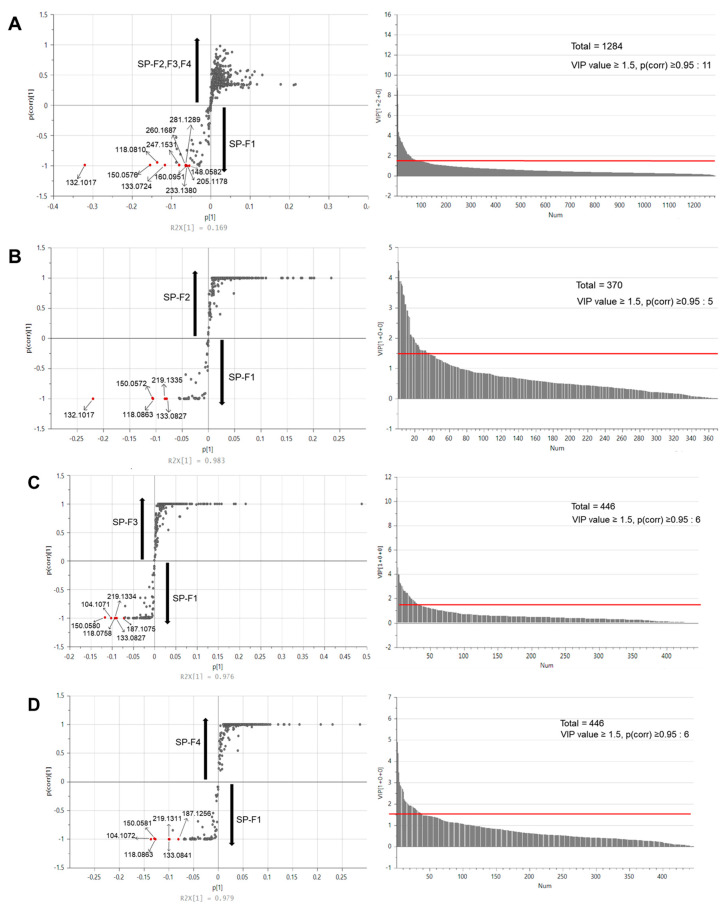
Multivariate statistical analyses for metabolites in the sub-subfractions of silkworm pupae protein hydrolysate (SP-Fs). Distinctive metabolites were screened based on S-plot and VIP generated by orthogonal partial least-squared discriminant analysis (OPLS-DA). Significantly different variables (*p* < 0.05) with VIP values over 1.5 were selected as featured metabolites in each fraction. Metabolomic analyses were conducted according to the following comparing groups: (**A**) SP-F1 vs. SP-F2 + SP-F3 + SP-F4; (**B**) SP-F1 vs. SP-F2; (**C**) SP-F1 vs. SP-F3; and (**D**) SP-F1 vs. SP-F4.

The S-plot derived from OPLS-DA determined the peptides or compounds with C2C12 myogenic differentiation-promoting activity. SP-F1, SP-F2, SP-F3, and SP-F4 were compared, and distinct compounds in SP-F1 (VIP value ≥ 1.5, *p* < 0.05) were screened (Figure 5). Overall, 11 (SP-F1 vs. SP-F2, SP-F3, and SP-F4), 5 (SP-F1 vs. SP-F2), 6 (SP-F1 vs. SP-F3), and 6 (SP-F1 vs. SP-F4) compounds were selected as potential peptides in silkworm pupae effective with C2C12 myogenic differentiation-promoting activity.

The distinct compounds in SP were tentatively identified to explain the myogenic differentiation effect. The identified compounds are indicated in Table 1 with their respective MS/MS fragments and MS error. The major amino acids that promote C2C12 myogenic differentiation in fraction F1, such as Ile and Val, were quantified using LC-MS/MS in the parallel reaction monitoring (PRM) mode. The content of Ile was 93.27 mg/g, and Val was 16.37 mg/g.

## 4. Discussion

Edible insects are a good source of bioactive peptides due to their advantage in terms of high protein content. Recently, the functional effects of edible insects were reported, such as antioxidant, anti-inflammation, and α-glucosidase inhibitory activity. However, the regulatory effect of edible insects on muscle was not reported. The present study used pupae protein hydrolysate to examine the effects on C2C12 cells’ myogenic differentiation.

Myogenesis involves muscle stem cells and progenitor cells to proliferate as myoblasts and differentiate into myotubes [29]. Activated satellite cells migrate from an undamaged muscle to a damaged muscle when cells are injured and then fuse to form new muscle fibers or repair damaged fibers together with existing fibers [30,31]. Prior to maturation into myofibrils, C2C12 myoblast cells express MyoD, an early marker for myogenic regulators, and further differentiate into myocytes [32]. During the early differentiation stage, myoblasts differentiate into mononucleated myocytes, and later on, myocytes undergo fusion and late differentiation to form myotubes. At each stage up to the formation of multinucleated myotubes, Myogenin and MyHC are the most representatively expressed myogenic regulatory factors [33]. Therefore, MyoD, myogenin, and MyHC can be used as markers of myogenesis, as exemplified by the marked expression of these three transcription factors [34].

To investigate C2C12 cells’ myogenic differentiation of pupae protein hydrolysate, we prepared the silkworm pupae (*Bombyx mori*) hydrolysate, tested the immunofluorescence using an MTT assay and Western blot assay, and finally, analyzed with LC-MS/MS to identify and quantify the compounds that were contained in pupae hydrolysate. SP yields at each step are similar to a previous study [8]. Moreover, the protein content of silkworm pupae after defatting and hydrolysis was 84.2 ± 2.17% and 74.7 ± 1.89%, respectively, consistent with Anootthato et al. [35]. It is considered that silkworm pupae contain a lot of water-soluble components. Additionally, the result of the degradation patterns of protein hydrolysis using a different pretreatment demonstrate that the position of the bands after each pretreatment is similar to that of [8], and the significant constituents exhibited similar mobilities on SDS-PAGE to those reported by [35]. As the pretreatment steps were conducted, a progressive decrease in the larger-sized protein with a concomitant increase in smaller-sized peptides was observed. After the immunofluorescence assay, the differentiation-promoting effect of SP on C2C12 cells was shown concentration-dependently by SP. These results suggest that SP promotes the differentiation of C2C12 myoblasts to myotubes compared to the control.

Previous studies investigated silkworm pupae-derived peptides’ antihypertension and antidiabetic activities [10,36]. For example, the silkworm pupae-derived dipeptides Gln-Pro-Gly-Arg, Ser-Gln-Ser-Pro-Ala, Gln-Pro-Pro-The, and Asn-Ser-Pro-Arg were identified as having high inhibitory activity against α-glucosidase (catalyzes the hydrolysis of starch to simple sugars), showing half-maximal inhibitory concentration values ranging from 20 to 560 μmol/L [10]. Moreover, yogurt incorporated with silkworm pupae-derived peptide enhanced the acidification, textural properties, and amino acid content [37]. Recently, Lee et al. [38] reported a protective effect of edible insect proteins extracted from *Protaetia brevitarsis* against oxidative stressed C2C12 myoblast cells. These edible insect proteins demonstrated a protective function by regulating the apoptotic process, potentially linked to the myogenesis of C2C12 cells. However, no studies were conducted on muscle growth with proteins from edible insects. Therefore, this study builds upon the existing knowledge base by suggesting that peptides from silkworm pupae could be utilized as a source of functional food for promoting muscle growth.

Muscle regeneration is a complex and strictly regulated process. When muscles are injured, satellite cells are activated and enter the cell cycle G1 phase to proliferate into myoblasts. MyoD-positive cells exit the cell cycle, myogenin begins to be expressed to initiate differentiation, and MyHC is expressed as the cells differentiate and fuse into myotubes. Activated satellite cells migrate from an undamaged muscle to a damaged muscle when cells are injured and then fuse to form new muscle fibers or repair damaged fibers together with existing fibers [30,31]. The expression level of MyoD, myogenin, and MyHC increased when C2C12 cells were treated with SP-F1. With SP-F1 treatment, a 1.5-fold increase in MyoD, a 2-fold increase in Myogenin, and a 3-fold change in MyHC were observed, indicating a significant promotion of myogenic differentiation.

In the metabolomic analysis, several *m*/*z* values were detected as distinctive markers and identified (Table 1). Val (*m*/*z* 118.0863), Ile (*m*/*z* 132.1017), Met (*m*/*z* 150.0581), Glu (*m*/*z* 148.0601), Asp-Phe (*m*/*z* 281.1127), Ala-Pro (*m*/*z* 187.1074), choline (*m*/*z* 104.1071), and unknown compounds were identified. According to previous studies, several of the compounds identified in this study are known for their ability to promote myogenesis. For example, branched-chain amino acids (BCAA), Val, and Ile are necessary for muscle growth maintenance through the production of intermediate metabolites in the tricarboxylic acid (TCA) cycle and gluconeogenesis [39,40]. Met is an essential amino acid that effects the expression of muscle growth factor genes and muscle structure genes [1]. However, other compounds such as Ala-Pro and Asp-Phe are yet to be investigated for the effect of promoting myogenic differentiation. From this study, we speculate that not only amino acids, but also dipeptides in the SP affect C2C12 myogenic differentiation, and further studies are needed.

## 5. Conclusions

This study investigated the effect of protein hydrolysates derived from edible insects on C2C12 myogenic differentiation. C2C12 cells were treated with the protein hydrolysate derived from silkworm pupae. The most effective protein hydrolysate subfractions were analyzed using LC-MS/MS-HESI-Orbitrap and multivariate statistical analysis to identify active peptides or compounds.

The SP effectively promoted C2C12 myogenic differentiation without cytotoxicity to C2C12 cells. The SP was then fractionated into four subfractions. Among them, SP-F1 was the most effective in promoting C2C12 myogenic differentiation (Figure 3) and significantly upregulated the expression of MyoD, myogenin, and MyHC (Figure 4). As a result of LC-MS/MS and multivariate statistical analysis, seven compounds, including choline, Glu, BCAA (Val and Ile), the essential amino acid Met, and the dipeptides Asp-Phe and Ala-Pro, stood out for their association with skeletal muscle development.

With increased interest being taken in the role of edible insects as a source of protein and their health and wellness benefits, additional research is needed to screen active compounds promoting myogenesis for muscle growth. The exploration in this study can be a useful foundation for upcoming research and development with edible insects.

## Figures and Tables

**Table 1:** Tentative identification of the key metabolites in the subfraction 1 of silkworm pupae protein hydrolysate (SP-F1) with promoting myogenic differentiation.

*m*/*z*	Tentative Identification	MS/MS Fragment	Error (ppm)
118.0863	Valine	72.0812, 55.0548, 118.0862	0.38
132.1017	Isoleucine	87.0967, 69.0703, 132.1316	−1.55
150.0581	Methionine	61.0112, 56.0500, 104.0529	−1.50
148.0601	Glutamic acid	84.0447, 56.0500, 102.0550	−1.58
281.1127	Asp-Phe	120.0806, 103.0543, 91.0544	−1.77
187.1074	Ala-Pro	70.0656, 116.0705, 84.0447	−1.60
104.1071	Choline	104.1070, 60.0813, 58.0656	1.05
219.1334	unknown	60.0449, 86.0967, 132.1016	
205.1180	unknown	80.0449, 72.0812, 118.0861	
247.1283	unknown	72.0812, 84.0447, 148.0601	-
160.1077	unknown	55.0548, 60.0813, 101.0598	-
260.1687	MS/MS not detected		-
233.1380	MS/MS not detected		-
133.0827	MS/MS not detected		-

## Data Availability

The data used to support the findings of this study can be made available by the corresponding author upon request.
